# Obituary

**Published:** 1868-02

**Authors:** 


### OBITUARY.

Deceased—February 27, 1868, at his residence, near Cleveland, Ohio, Dr. Edward Taylor, aged 57 years.

Dr. Taylor was born near Bainbridge, Ross County, Ohio, which place might very properly be called the starting point of the following pioneers of the profession in the Mississippi Valley: Drs. John, James and Chapin A. Harris, and Dr. Jones, formerly of Dayton, Ohio; Dr. Wamphler, of Illinois; Dr. Church, of Baltimore; Drs. Joseph, James, and Irwin Taylor.

In 1830, and 1831, Edward Taylor pursued the study of Dentistry under the special direction of his brothers, Joseph and James, and commenced the practice in the latter part of 1831, spending the first winter of his professional career in some of the small towns along the Mississippi river. His first summer's practice was in Cynthiana, Ky., at which place he rendered very efficient service during the prevalence of the Cholera.

In 1833 he settled in Springfield, Ohio, where, by close attention to his practice and prosecution of medical studies, he soon won the confidence of the public. After a practice here of some two years, owing to ill-health and the loss of his wife, he left; and after spending some two years or more in travel and practice at Maysville, Ky., he finally settled in Natchez, Mississippi. At this place he soon secured a lucrative and pleasant practice, and remained for several years, his health evidently improved by the mild climate.

In 1844 or 1845, he opened an office in Louisville, Ky., intending here to spend the remainder of his professional life, but was soon induced to join his brother James in this city, where for some two years or more he maintained a high stand in his profession and made many warm friends. An attack of Cholera in 1847, with an enfeebled constitution, soon convinced him that the arduous duties of an office practice would render restoration to health impossible; and with the feeling that an out-door active life would be best for him, he engaged in a manufacturing business, and in the course of a couple of years recovered much of his wasted energies; but his tastes and inclinations leading him to horticulture and country life, and with a view of enjoying a more bracing atmosphere on the lake shore, he selected Cleveland as his residence, in the neighborhood of which he purchased a farm, and at once with much zest entered into the culture of fruit and nursery stock.

For the last few years his rheumatic and dyspeptic difficulty has been gradually encroaching on his constitution, so that for the last three months he was unable to leave his room and he gradually passed away with strong faith and firm trust in Christ as his Savior.

Dr. Taylor studied Dentistry as a specialty of medical science, and his early attainments were such in both directions that not only the Baltimore College of Dental Surgery, soon after its organization, conferred on him the honorary degree, D. D. S.; but he received from one of our regular Medical Colleges of the West the title of M. D. The early numbers of the REGISTER will show that he was a clear and accurate thinker, a forcible and logical writer, and ever devoted to the best interest of the profession. No man ever brought to the profession more unswerving integrity and a higher sense of professional honor.

Drs. Chapin A. and James Harris, although not residents of Bainbridge, yet took their first lessons in Dental Practice from Dr. John Harris while residing at that place. T.

